# Successful treatment of pathologic femoral shaft fracture associated with large arteriovenous malformations using a 3-dimensional external fixator and teriparatide: a case report

**DOI:** 10.1186/s12893-019-0498-4

**Published:** 2019-04-05

**Authors:** Akihiko Takeuchi, Hidenori Matsubara, Norio Yamamoto, Katsuhiro Hayashi, Shinji Miwa, Kentaro Igarashi, Hiroyuki Inatani, Hiroyuki Tsuchiya

**Affiliations:** 1 Department of Orthopaedic Surgery, Graduate School of Medical Science, Kanazawa University, 13-1 Takara-machi, Kanazawa-shi, Ishikawa-ken 920-8641 Japan; 2 Department of Orthopaedic Surgery, Fukui-ken Saiseikai Hospital, 7-1 Funabashi Wadanakacho, Fukui-shi, Fukui-ken 918-8503 Japan

**Keywords:** Arteriovenous malformation, Pathologic fracture, Taylor spatial frame, Teriparatide

## Abstract

**Background:**

Arteriovenous malformations (AVMs) are rare congenital vascular lesions associated with early quiescence, late expansion, and, ultimately, infiltration and destruction of local soft tissue and bone. The extremities are a common location. Incidence of bony involvement by AVM has been reported as high as 31%. However, there are few reports on management of pathologic fracture associated with AVM. Teriparatide is a recombinant parathyroid hormone (PTH) analogue consisting of the 1–34 fragment of PTH. Recently, some reports have shown the ability of teriparatide to improve fracture healing. Here, we present a case of pathologic femoral shaft fracture associated with large AVMs that was treated successfully by external fixation and teriparatide.

**Case presentation:**

A 68-year-old Japanese woman, previously diagnosed as having large AVMs, sustained a right femoral shaft fracture due to a fall. At the time of admission, she presented with massive swelling and venous varicosities of the right thigh. Plain radiography of the right thigh revealed femoral shaft fracture with bony erosion and calcification of soft tissue. We planned closed reduction and intramedullary nailing with a unilateral external fixator following embolization of the feeding artery. However, closed reduction using the fracture table was difficult. When we attempted open reduction, massive bleeding (1000 mL) after incision of subcutaneous tissue occurred. Finally, we carefully applied a Taylor Spatial Frame. Fracture displacement was corrected successfully and bony union was obtained with administration of teriparatide 15 months after the initial surgery. The patient is able to walk using 1 cane.

**Conclusion:**

We present the first report of pathologic fracture associated with large AVMs that achieved bony union using a 3-dimensional external fixator and teriparatide.

## Background

Arteriovenous malformations (AVMs), formerly referred to as arteriovenous hemangiomas, are rare congenital vascular lesions. The International Society for the Study of Vascular Anomalies classified these vascular anomalies into either vascular tumors (mostly hemangiomas) or vascular malformations. Vascular malformations are further divided into capillary malformations, venous malformations, lymphatic malformations, arteriovenous malformations (AVMs), and combined lesions. AVMs contain abnormal shunts between arteries and veins with high-flow malformations [1]. AVMs most commonly occur in the extremities, pelvis, midface, and oral cavity, and are associated with early quiescence, late expansion, and, ultimately, infiltration and destruction of local soft tissue and bone [2]. AVMs can also cause local destructive skeletal change and pathologic fracture [3, 4]. There are several treatment options for AVM, including conservative therapy with fitted pressure garments [5], systemic corticosteroids [2], embolization [6], radiation [7], sclerotherapy [8], and surgical removal [3], or a combination of these modalities [9, 10]. Incidence of bony involvement by AVM has been reported to be 20 to 31% [3, 11]. However, there are few reports on management of pathologic fracture associated with AVM [4].

Teriparatide, a parathyroid hormone (PTH) analogue, is a synthetic polypeptide hormone consisting of the 1–34 fragment of PTH, which is used in treatment of osteoporosis [12]. Recently, some reports have shown the ability of teriparatide to improve fracture healing [13, 14].

Here, we present a case of pathologic femoral shaft fracture associated with large AVMs that achieved bony union using a 3-dimensional external fixator and teriparatide.

## Case presentation

**Fig. 1:**
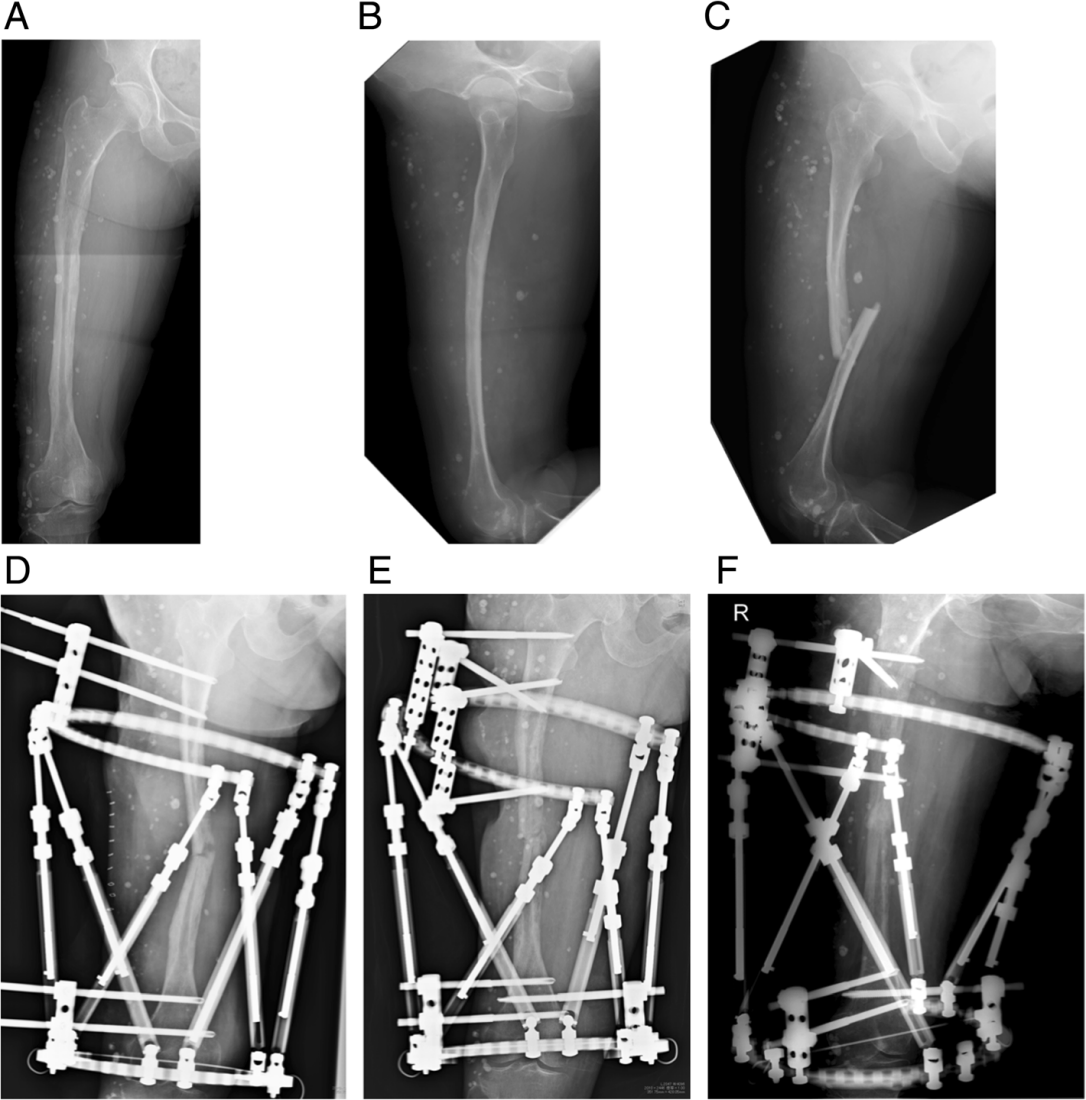
Pre- and postoperative radiographs of the right thigh. Anterolateral (**a**) and lateral (**b**) radiographs taken at the first visit at 63 years of age reveal soft tissue swelling containing numerous phleboliths and bony erosion in the femoral shaft. **c** Radiograph taken on admission at 68 years of age reveals displaced pathologic right femoral shaft fracture. **d** Radiograph taken immediately after the first surgery reveals partial reduction of the fracture using a Taylor Spatial Frame. **e** Radiograph taken immediately after the second surgery reveals replacement of the proximal half-pins and complete reduction of the fracture. **f** Radiograph taken 5 months after the second surgery reveals bony union. One of the proximal half-pins was removed 1 month after the second surgery due to loosening

A 68-year-old Japanese woman, previously diagnosed as having large AVMs, sustained a right femoral shaft fracture due to a slip and fall from standing. Bruising of the right thigh and limping were noted since 3 years of age. She was diagnosed as having AVMs at 63 years of age (Fig. 1a and b), but did not receive any treatment.

**Fig. 2:**
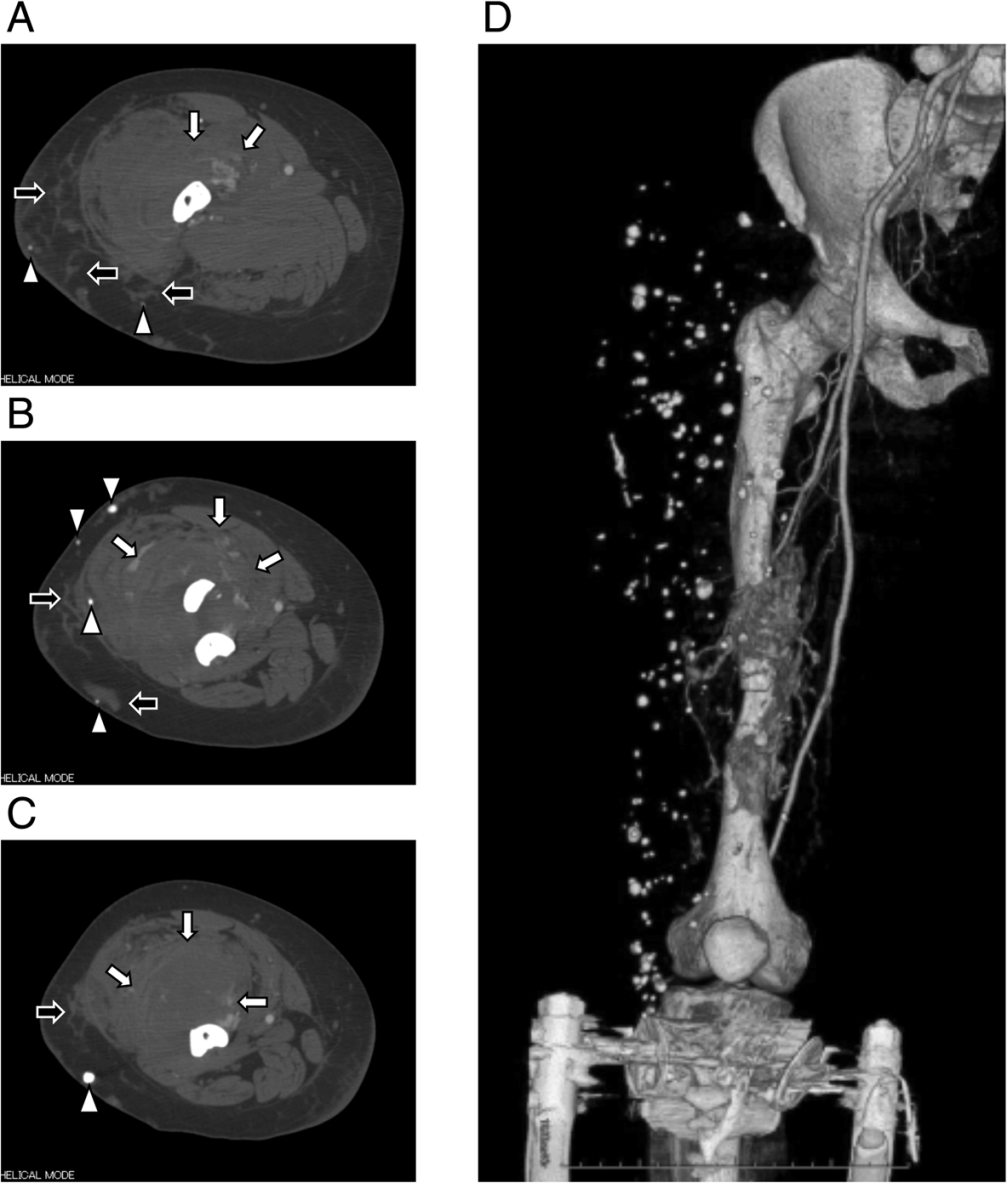
Preoperative enhanced computed tomography scans show large arteriovenous malformations (AVMs) in the quadriceps femoris (white arrows), which are partially present subcutaneously (black arrows). Numerous phleboliths (white arrowheads), massive bony erosion, and very narrow intramedullary canal are also observed (**a**: proximal site of fracture; **b**: fracture site; **c**: distal site of fracture). **d** Three-dimensional computed tomography angiography scan shows the AVMs are fed by the branches of the deep and superficial femoral artery

At the time of admission, she presented with massive swelling and venous varicosities of the right thigh. Plain radiography of the right thigh revealed fracture of the femoral shaft with bony erosion and numerous phleboliths (Fig. 1c). Computed tomography showed enhanced AVMs and phleboliths in the quadriceps femoris and the hypotrophy of the femur. In addition, the intramedullary canal was very narrow (Fig. 2a, b and c), so intramedullary nailing would be difficult. Three-dimensional computed tomography angiography showed the AVMs were fed by the branches of the deep and superficial femoral artery (Fig. 2d). We planned closed reduction and intramedullary nailing using elastic nails fixed to a monotube unilateral external fixator.

**Fig. 3:**
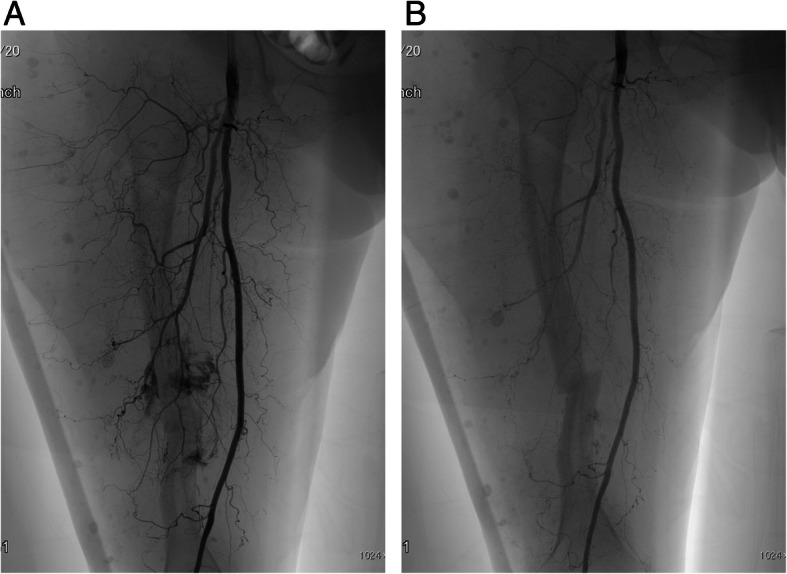
**a** Preoperative angiogram shows feeding from the deep femoral artery. **b** After embolization, the niduses of the arteriovenous malformations were reduced

The day before surgery, embolization of the feeding artery was performed (Fig. 3a and b). We attempted closed reduction of the fracture using the fracture table, but the procedure was difficult. When we attempted open reduction, however, massive bleeding (1000 mL) after incision of subcutaneous tissue occurred within several minutes. Therefore, we closed the wound immediately. Although an additional procedure carried the risk of further bleeding, we carefully applied a Taylor Spatial Frame (TSF) and performed acute correction (Fig. 1d). Total blood loss was 2300 mL and she was transfused with 23 units of red cell concentrate, 10 units of fresh-frozen plasma, and 20 units of platelet concentrate. She was subsequently transferred to the intensive care unit and received 8 more units of red cell concentrate.

**Fig. 4:**
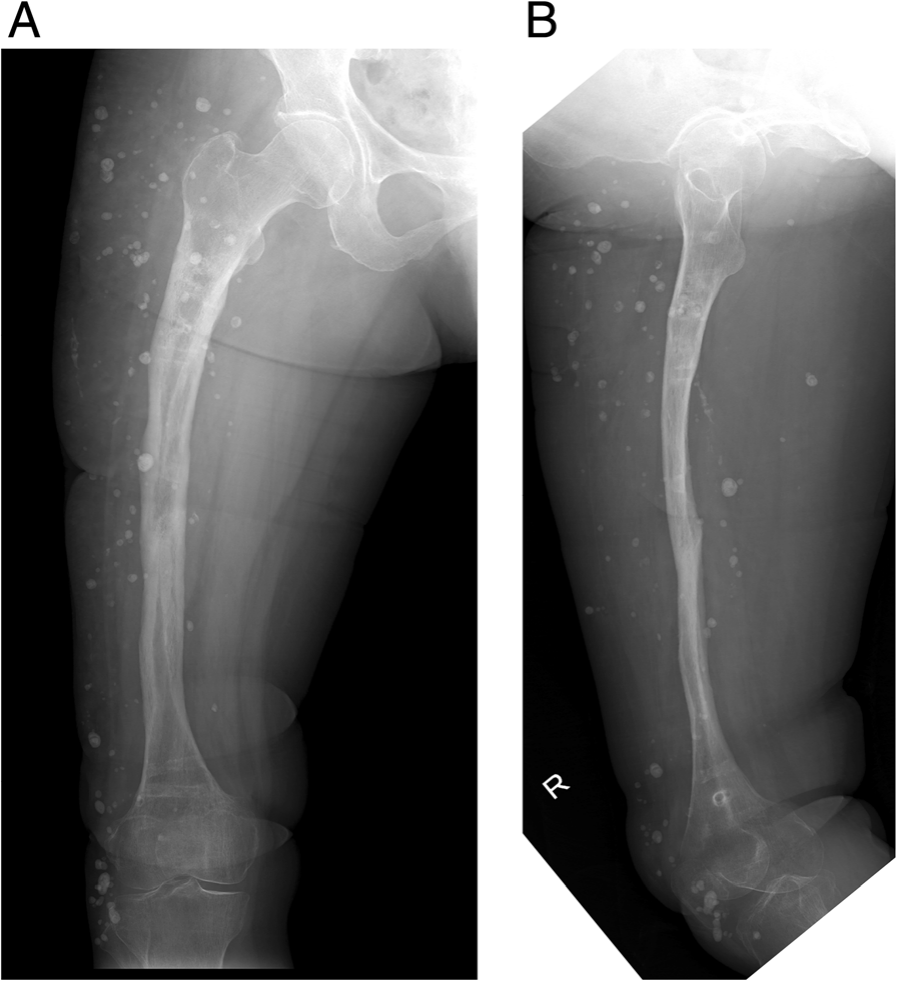
Anterolateral (**a**) and lateral (**b**) radiographs taken 24 months after the second surgery show complete bony union. The Taylor Spatial Frame was removed 11 months after the second surgery

Her postoperative course was uneventful. Residual displacement of the fracture was gradually corrected over 3 weeks. Teriparatide (20 μg daily) was initiated. After 10 weeks, loosening of the proximal half-pins and re-displacement of the fracture occurred. Thus, we performed replacement of the proximal half-pins (Fig. 1e). Eventually, bony union was obtained 13 months after the second surgery (Fig. 1f), and the TSF was removed 2 months later. She began weight bearing gradually with Sarmiento type functional brace [15]. Teriparatide was administrated for 24 months. Radiographs taken 24 months after surgery showed the facture was completely united (Fig. 4a and b). The patient became fully ambulatory with Sarmiento type functional brace using 1 cane.

## Discussion and conclusions

The present case demonstrates that pathologic fracture associated with huge AVMs can be managed by minimally invasive fixation using a TSF and administration of teriparatide. The problems in this case were that the large AVMs existed in a wide anterolateral site of the femur and that the femur was very thin. To achieve stability, fixation using a locking plate or intramedullary nailing was necessary. However, open reduction and internal fixation carried a risk of massive bleeding. Also, the intramedullary canal was very narrow, so intramedullary nailing was extremely difficult.

AVMs are usually present at birth, progress gradually, and sometimes become clinically evident later in life with pain, swelling, ulceration, leg length discrepancy, bleeding, and venous varicosities [3, 16]. Breugem et al. reported that in 90 patients with vascular malformations of the lower limbs, 18 (20%) had bony involvement. They mentioned that these malformations should be treated based on their own merits using a multidisciplinary approach (compression stockings and analgesia, surgical resection and/or intralesional transarterial embolization, and amputation). Although massive intraosseous involvement results in decreased bone density and increased risk of fracture, they warned that surgical treatment is often associated with profuse bleeding, incomplete resection, and local recurrence [3].

There are few reports on management of fracture associated with AVM. Jonczyk et al. reported the case of a 19-year-old man with pathologic subtrochanteric left femoral fracture who had extensive AVM of the left thigh. They treated the fracture with intramedullary nailing following embolization. The fracture was closely reduced on a traction table. Even though this patient had a risk of massive bleeding, he required only 2 units of packed red blood cells intraoperatively followed by 2 more units on the fourth postoperative day. The fracture was eventually united 9 months after surgery [4].

There are some reports of patients with Klippel-Trenaunay syndrome (KTS) who suffered femoral shaft fracture [17, 18]. KTS is characterized by capillary malformations and venous anomalies with bony and soft tissue hypertrophy in 1 or more limbs [19]. Gupta et al. reported a patient with KTS and femoral shaft fracture that was treated by external fixation following closed reduction. In this case (a 12-year-old girl), the elastic intramedullary nail was quite difficult to insert due to poor bone quality and risk of cortical perforation. The fracture eventually united 4.5 months after surgery [18]. Although the present case lacked the diagnostic criteria for KTS, a more difficult situation existed in that the skin incision involved the AVMs, the femoral shaft was very thin, and closed reduction was difficult. Finally, we applied a TSF on the femur and reduction was obtained.

However, we risked delayed union or refracture due to the very thin femur due to the extensive AVM surrounding the femur. It was extremity difficult to perform the additional bone graft due to the risk of massive bleeding. Thus, we initiated teriparatide beginning 2 weeks after surgery. Teriparatide, a recombinant PTH analogue, is a synthetic polypeptide hormone consisting of the 1–34 fragment of PTH, which is used in treatment of osteoporosis [12]. Borges et al. reported enhanced fracture healing after administration of teriparatide in an 84-year-old woman who sustained a fall-related transtrochanteric fracture [20]. Kim et al. evaluated the evidence of teriparatide for fracture healing and functional recovery in patients with osteoporosis as a systemic review. They reported that teriparatide yielded positive effects on radiographic bone healing in 6 studies, but was not associated with better radiographic outcome in 3 studies. They concluded that teriparatide provides selective advantages to fracture healing or functional recovery in management of osteoporotic fractures [13]. Bhandari et al. performed a placebo-controlled phase 3 study to evaluate the effect of teriparatide on fracture healing. Although the sample size was small, teriparatide did not decrease the risk of revision surgery, improve radiographic signs of fracture healing, or decrease pain compared with placebo [21]. In the present case, we believe teriparatide contributed to healing of the pathologic fracture with a very thin femoral shaft because bony union was obtained with massive callus formation, which allowed for full weight bearing.

The TSF is a unique external fixation system that provides excellent fracture reduction using a computer program and noninvasive fixation of the fracture site [22]. This contributes to a minimally invasive procedure in patients with AVM.

In conclusion, we encountered a case of pathologic femoral shaft fracture associated with huge AVMs that achieved bony union using a 3-dimensional external fixator and teriparatide. Although treatment of pathologic fracture due to AVM is challenging, a minimally invasive, multidisciplinary approach may offer the best result.

## Abbreviations

AVM: Arteriovenous malformation
KTS: Klippel-Trenaunay Syndrome
PTH: Parathyroid hormone
TSF: Taylor Spatial Frame
